# University Law Suit

**Published:** 1852-03

**Authors:** 


### THE WESTERN JOURNAL.

—

THIRD SERIES.—VOL. IX.—NO. III.

—

LOUISVILLE, MARCH 1, 1852.

---

### UNIVERSITY LAW SUIT.

It is known to our readers that for nearly a year past, a suit under the new city charter has been pending between the city of Louisville and the Board of Trustees of the University. By a provision of the new charter, the University was placed under a new Board of Trustees to be elected by the people, one half going out every year; but as the constitutionality of this clause was doubted by many members of the Legislature, the question was submitted for adjudication to the Jefferson Circuit Court. We take much pleasure in announcing that the case has been decided, and that the decision is in favor of the Trustees. The administration of the school, consequently, remains in the hands where it was.

The friends of the school will rejoice at this settlement of a question which was believed by many to jeopard its prosperity. To say nothing more, it cannot be considered bad policy to "*let well enough alone.*" The institution has certainly done well under its present administration; its friends could hardly have hoped that it would have done better; and the policy of tampering with its organization, under such circumstances, may well be questioned.

